# Novel chromosome segment substitution lines derived from *japonica* cultivar ‘Yukihikari’ in the genetic background of ‘Joiku462’ cultivar and identification of quantitative trait loci for heading date and grain quality

**DOI:** 10.1270/jsbbs.24058

**Published:** 2025-06-18

**Authors:** Kiyoaki Kato, Shinya Munekata, Toshiro Watanabe, Takashi Sato, Yusuke Hosokawa

**Affiliations:** 1 Department of Agro-Environmental Science, Obihiro University of Agriculture and Veterinary Medicine, Nishi 2-11 Inada, Obihiro, Hokkaido 080-8555, Japan; 2 Rice Breeding Group, Kamikawa Agricultural Experiment Station, Local Independent Administrative Agency, Hokkaido Research Organization, Minami 1-5, Pippu, Hokkaido 078-0397, Japan; 4 Plant Nutrition Laboratory, Research Faculty of Agriculture, Hokkaido University, Sapporo, Hokkaido 060-8689, Japan

**Keywords:** rice, chromosome segment substitution lines, QTL cluster, apparent amylose content, protein content, grain size, elemental content

## Abstract

In this study, we mapped quantitative trait loci (QTLs) associated with heading date and grain quality traits in a novel set of 44 chromosome segment substitution lines (CSSLs) derived from closely related rice (*Oryza sativa* L. ssp. *japonica*) cultivars ‘Yukihikari’ (good grain quality) and ‘Joiku462’ (superior eating and high grain appearance qualities). Days to heading (DTH), apparent amylose content (AAC), protein content (PC), thousand brown-grain weight (TBGW), brown grain length (BGL), brown grain width (BGWI), brown grain thickness (BGT), and the contents of 12 mineral elements (S, P, Mg, Ca, K, Mo, Cu, Zn, Mn, Fe, Sr, and Si) in polished rice were evaluated in 44 CSSLs grown in two different environments. We identified 78 QTLs, including 8, 7, 8, 8, 19, 10, and 10 for DTH, AAC, PC, TBGW, BGL, BGWI, and BGT, respectively, and 2, 1, 3, and 2 for S, Mo, Cu, and Zn contents, respectively. Several QTLs were observed in the same region, forming 17 clusters on chromosomes 1–10. These QTLs can facilitate gene isolation and breeding to develop rice cultivars with optimum heading time and improved grain quality.

## Introduction

Rice (*Oryza sativa* L.) is one of the most important staple crops, feeding almost half the world’s population. Therefore, an improvement in rice grain yield is required to ensure food security. In addition to rice yield, grain quality has received particular attention from consumers, farmers, seed producers, and the food industry (Champagne *et al.* 1999, Fitzgerald *et al.* 2009). Therefore, breeding elite rice cultivars with high yields and quality is a major goal for geneticists and breeders (Bao 2014).

Heading date is an important agronomic trait of rice and determines the regional and seasonal adaptability of rice varieties and significantly impacts grain yield and quality (Hori *et al.* 2012, Xue *et al.* 2008, Zhou *et al.* 2021). Moreover, it is crucial to breed rice cultivars with optimum heading dates suitable for cropping areas in which these cultivars can be used to maximize rice production. More than 40 different genes (>40 gene loci) regulating flowering time in rice have been identified using several approaches, including map-based cloning, reverse genetics using T-DNA or transposon tagging lines, and control of target gene expression via RNAi and overexpression (*reviewed by* Vicentini *et al.* 2023).

Grain quality is a complex quantitative trait determined by multiple characteristics, including the physical appearance, milling quality, nutritional value (grain components), aroma, and cooking and eating qualities of rice. Many genes have been implicated in the control of grain quality traits (Li *et al.* 2022). The eating quality of rice is affected by many agronomic characteristics, including heading date, grain size and weight, grain number per panicle, and spikelet fertility (Iijima *et al.* 2019, Juliano *et al.* 1993).

Apparent amylose content (AAC) is a major factor determining eating and cooking qualities in rice, particularly the sensory properties of cooked rice (Bhattacharya 1985, Fitzgerald *et al.* 2009). Amylose has a straight-chain structure and is one of the two components that form starch granules in rice grains (Peng *et al.* 2021). AAC is positively correlated with the overall hardness of rice and water absorption, indicating that an appropriate reduction in AAC can improve eating and cooking qualities (Li and Gilbert 2018). Based on their AAC, rice varieties can be classified as waxy rice (1 to 2%) and extremely low (2 to 12%), low (12 to 20%), intermediate (20 to 25%), and high (>25%) AAC rice. The waxy (*Wx*) gene, which encodes granule-bound starch synthase I, controls amylose synthesis in the rice endosperm (Hanashiro *et al.* 2008, Okagaki 1992). In addition to the *Wx* gene, which is directly involved in amylose synthesis, dozens of genes implicated in the transcriptional and post-transcriptional regulation of the *Wx* gene have been cloned (*reviewed by* Ren *et al.* 2023).

Grain protein content (PC) is another factor that affects the eating and cooking qualities of rice (Wang *et al.* 2017). The content and composition of rice grain protein determine the surface hardness of cooked rice (He *et al.* 2021, Zhong *et al.* 2021). PC affects the digestibility and flavor of cooked rice (Syahariza *et al.* 2013). Rice with a low PC has a more desirable flavor than in rice with a high PC (Champagne *et al.* 2004). A negative correlation exists between PC and the eating quality of rice (Hori *et al.* 2016). However, PC directly affects the nutritional quality of rice. Therefore, designing a reasonable regulatory strategy for increasing PC in rice can help maintain its eating and cooking qualities while enhancing its nutritional quality (Ren *et al.* 2023). PC is quantitatively inherited and extensively affected by environmental factors (Chattopadhyay *et al.* 2019, Kinoshita *et al.* 2017, Pradhan *et al.* 2019). Numerous quantitative trait loci (QTLs) for PC have been previously reported, which has been repeatedly confirmed (Chattopadhyay *et al.* 2019, Cheng *et al.* 2013, Kashiwagi and Munakata 2018, Lou *et al.* 2009, Tan *et al.* 2001, Wang *et al.* 2007, Yang *et al.* 2015, Ye *et al.* 2010, Zheng *et al.* 2011, 2012). Hence, QTLs dissected for PC frequently vary based on the structure of a given population or the environmental conditions. A few cloned genes have been associated with PC in natural rice populations (Peng *et al.* 2014, Yang *et al.* 2019).

Complex interactions between major and minor QTLs regulate grain size and shape, which, in turn, determine rice yield and grain quality (Li *et al.* 2020). In Japan, brown rice grains are mechanically sieved through a mesh width of 1.7–2.0 mm, depending on cultivar and location. This sieving method yields two fractions consisting of thick (>1.7 mm) and thin (<1.7 mm) grains, with the thick grains being more marketable than the thin grains (Kinoshita *et al.* 2017). Recently, 2.0 mm mesh has increasingly been used to separate thin brown rice grains, making it essential to use rice cultivars that produce brown rice of thickness >2.0 mm. Therefore, numerous genetic studies have been conducted to map QTLs associated with grain shape in rice. Over the past few decades, thousands of QTLs have been identified in various mapping populations (Dong *et al.* 2018, Hu *et al.* 2018, Kinoshita *et al.* 2017, Ying *et al.* 2018, Zhang *et al.* 2020). Of these, nearly 200 have been cloned as functional genes related to rice grain size with direct or indirect roles (*reviewed by* Jiang *et al.* 2022).

Polished rice is a staple food consumed worldwide. However, it contains limited amounts of mineral elements. Therefore, developing rice cultivars with increased mineral element contents is the most cost-effective and efficient approach for alleviating human malnutrition and nutrient deficiencies. Genes responsible for mineral element content in rice grains, mainly brown rice, have been mapped using QTLs. More than 200 QTLs have been identified (*reviewed by* Das *et al.* 2020). However, relatively little is known about the QTLs associated with the elemental content in polished rice.

In our previous studies (Kinoshita *et al.* 2016, 2017), QTLs associated with grain quality and yield-related traits were mapped in recombinant inbred lines (RILs) derived from closely related rice (*O. sativa* L. ssp. *japonica*) cultivars, ‘Yukihikari’ (good eating quality, released in 1981) and ‘Joiku462’ (superior eating and high grain appearance qualities, released in 2009). In these studies, days to heading (DTH), AAC, PC, brown grain length (BGL), brown grain width (BGWI), brown grain thickness (BGT), thousand brown-grain weight (TBGW) per plant, and nine yield-related traits were evaluated in 133 RILs grown in four different environments in Hokkaido, near the northernmost limit for rice paddy cultivation. A total of 72 QTLs were detected, including five for DTH, three for AAC, eight for PC, seven for TBGW, two for BGL, four for BGWI, and seven for BGT, on chromosomes 1, 2, 3, 4, 6, 7, 8, 9, 11, and 12, using 178 molecular markers. However, it is necessary to evaluate these putative QTLs to generate rice cultivars with improved grain quality and yield. Furthermore, it is crucial to identify beneficial alleles in previously cloned genes and novel QTLs of target traits for optimizing crop genomes through the accumulation of beneficial alleles (Mascher *et al.* 2019, Varshney *et al.* 2021). Takano *et al.* (2014) identified 1,842 non-synonymous nucleotide polymorphisms in 948 genes and 141 protein-altering indels in ‘Yukihikari’ and ‘Joiku462’. These functional mutations are potential causal genes associated with the quantitative traits.

Chromosome segment substitution lines (CSSLs) are advanced backcrossed populations in which single chromosomal segments derived from a donor are substituted in the genetic background of a recurrent cultivar. CSSLs are useful genetic materials for dissecting complex agronomic traits, such as heading date, yield components, and grain quality in rice, with high sensitivity using fewer plants than other genetic mapping populations (*reviewed by* Balakrishnan *et al.* 2019).

In the current study, we constructed a novel set of CSSLs derived from the *japonica* cultivars ‘Yukihikari’ and ‘Joiku462’, and backcrossing ‘Yukihikari’ as the donor and ‘Joiku462’ as the recipient. A total of 167 CSSLs were constructed. Among these, 44 CSSLs were selected as the minimum set required to cover the donor genome. QTLs determining DTH, AAC, PC, TBGW, BGL, BGWI, and BGT and 12 mineral element contents were analyzed using these 44 CSSLs.

## Materials and Methods

### Development of CSSLs

**Fig. 1.**
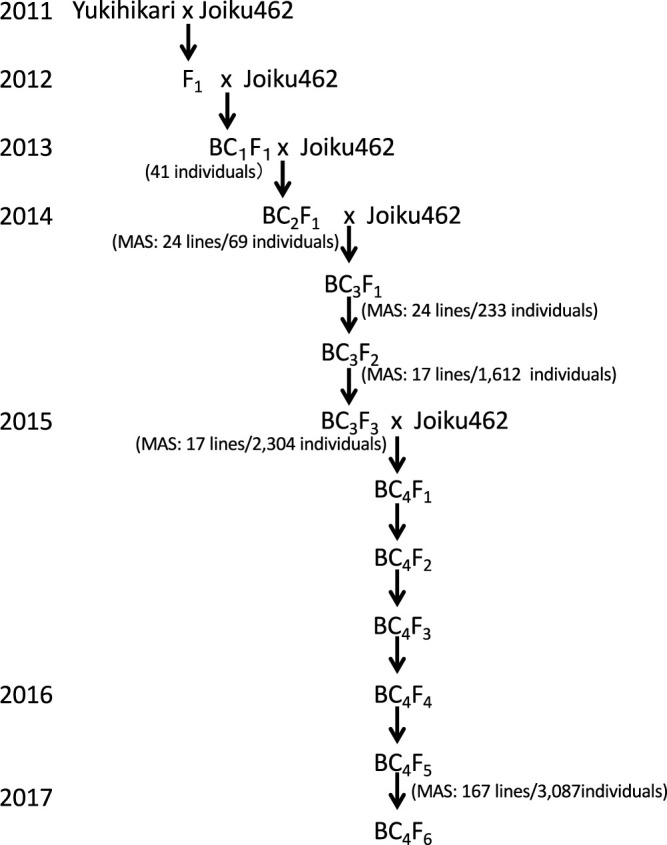
Schematic of the development of chromosome segment substitution lines (CSSLs) carrying ‘Yukihikari’ chromosome segments in a ‘Joiku462’ genetic background. The numerator and denominator in parentheses indicate the number of plants selected and investigated by marker-assisted selection (MAS), respectively. A total of 7,305 plants were used to develop the CSSLs.

The process involved in the development of CSSLs is illustrated in Fig. 1. F_1_ plants derived from a cross between ‘Yukihikari’ and ‘Joiku462’ were backcrossed to ‘Joiku462’ to produce 41 BC_1_F_1_ plants. Of 69 resulting BC_2_F_1_ plants, 24 were selected using the marker-assisted selection (MAS) technique and backcrossed to ‘Joiku462’ to generate the BC_3_F_1_ generation. Of 233 BC_3_F_1_ plants genotyped using DNA markers, 24 BC_3_F_1_ plants were screened to produce the next generation via self-pollination. In total, 1,612 BC_3_F_2_ plants were genotyped using DNA markers, and 17 BC_3_F_2_ individuals were screened using MAS to generate the next generation via self-pollination. Subsequently, 2,304 BC_3_F_3_ plants were genotyped using DNA markers, and 17 BC_3_F_3_ individuals were screened as pre-CSSLs for the homozygous substitution of one large or multiple chromosome segment(s) to cover almost all donor chromosomes using MAS. To reduce the size of the target segment, residual non-target fragments, or both derived from the donor, the 17 selected BC_3_F_3_ individuals were backcrossed to ‘Joiku462’ to generate BC_4_F_1_ generation. In total, 5,970 BC_4_F_2_ plants were self-pollinated to generate BC_4_F_3_ plants. RILs were developed from BC_4_F_3_ to BC_4_F_5_ using the single-seed descent method. In total, 3,087 BC_4_F_5_ plants were genotyped using DNA markers, and 167 individuals were screened for homozygous CSSLs using MAS.

### Analyses using InDel markers, cleaved amplified polymorphic sequence markers, and derived cleaved amplified polymorphic sequence markers

In the current study, we used 163 InDel markers (Kinoshita *et al.* 2016). In addition, we used seven cleaved amplified polymorphic sequence (CAPS) markers and eight derived CAPS (dCAPS) markers (Kato and Hirayama 2021, Kinoshita *et al.* 2016). DNA extraction, polymerase chain reaction, restriction enzyme digestion, and gel electrophoresis were performed as previously described (Kato and Hirayama 2021, Kinoshita *et al.* 2016).

### Field trials

In the first trial, rice seeds were sown in a greenhouse at the Obihiro University of Agriculture and Veterinary Medicine on April 25, 2019. Seedlings aged 36 days were transplanted into the paddy fields of Kamikawa Agricultural Experiment Station (KAES, Pippu, 43° 51ʹ N, 142° 48ʹ E) on May 31, 2019. In the second trial, seeds were sown in a greenhouse at KAES on May 1, 2020. Seedlings aged 28 days were transplanted into the paddy fields of KAES on May 29, 2020. All seedlings were transplanted at a density of one plant per hill and a spacing of 30 × 15 cm (22.2 plants/m^2^). Forty plants from each parental line and CSSL were grown in triplicates. Plants were fertilized with 8 kg N/10a, 9.7 kg P_2_O_5_/10a, and 6.9 kg K_2_O/10a.

### Evaluation of agronomic traits

DTH was defined as the number of days from sowing to the stage with more than 50% of plants exhibiting heading. To measure TBGW, grains collected from more than eight plants of each parental line or CSSL were pooled, air-dried to a 15–16% moisture content, and weighed. The combined weight of two samples of 500 randomly chosen brown rice grains per line was defined as TBGW. BGL, BGWI, and BGT were measured in 500 randomly selected brown rice grains from each line using a Satake Grain Scanner (RGQI10B; Satake, Hiroshima, Japan) and averaged. More than 50 g of brown rice was polished to a yield of approximately 90% in a rice mill (SKM5B(1); Satake, Hiroshima, Japan). The AAC of polished rice from each line was evaluated as described (Juliano *et al.* 1965), and the duplicated PC of polished rice from each line was determined using an Infratec™ 1241 Grain Analyzer (Foss, Hillerød, Denmark).

### Mineral element content analyses

Seeds of 44 CSSLs and parental lines grown at KAES for 2 years were used for the analysis. Approximately 10 g of 90%-polished rice was crushed using a multibead shocker (Yasui Kikai, Osaka, Japan). Plant samples (100 mg each) were digested using 2 mL of 61% (w/v) nitric acid (HNO_3_) (EL grade; Kanto Chemical, Tokyo, Japan) at 110°C in a DigiPREP apparatus (SCP Science, Montreal, Canada) for approximately 2.0 h until the solution had almost completely evaporated. After the samples had cooled, 0.5 mL H_2_O_2_ (semiconductor grade; Santoku Chemical, Tokyo, Japan) was added, and the samples were heated to 110°C for an additional 20 min. Once digestion was complete, the tubes were cooled, and the samples were reconstituted to a volume of 10 mL by adding 2% (w/v) HNO_3_ in ultrapure water. The concentrations of S, P, Mg, Ca, K, Mo, Cu, Zn, Mn, Fe, Sr, and Si (acid-soluble) were measured by using an inductively coupled plasma mass spectrometer (ELAN DRC-e; PerkinElmer, Waltham, MA, USA). External calibration standards containing these elements were measured every 10 samples.

### Detection of QTLs

QTL analysis was performed on CSSLs that showed traits significantly different from those of the recurrent parent ‘Joiku462’, based on Dunnett’s multiple comparison tests at a 95% confidence interval (*P* < 0.05). Subsequently, QTLs were assigned to the chromosomal regions of these CSSLs. A QTL detected in a single CSSL was regarded as being located on non-overlapping chromosome segments, whereas QTLs detected in multiple CSSLs were considered to reside on overlapping chromosome segments. The name of each QTL consists of information regarding the abbreviation of its trait followed by ‘b’ to distinguish them from the QTLs identified in our previous study using RILs (Kinoshita *et al.* 2017) and chromosome number.

### Candidate causal gene detection

The physical position of each QTL was determined based on the position of the flanking InDel, CAPS, or dCAPS markers, and genes physically located within the QTLs for agronomic and grain mineral content were considered candidate genes. Genes annotated with functions related to agronomic traits, metal transport, and homeostasis were compiled, and their physical positions were determined using the Genome Browser of the Rice Annotation Project Database (https://rapdb.dna.affrc.go.jp/). The annotations and functions attributed to different candidate genes were downloaded from Oryzabase (https://shigen.nig.ac.jp/rice/oryzabase/gene/advanced/search).

## Results

### Characterization of the CSSLs

**Fig. 2.**
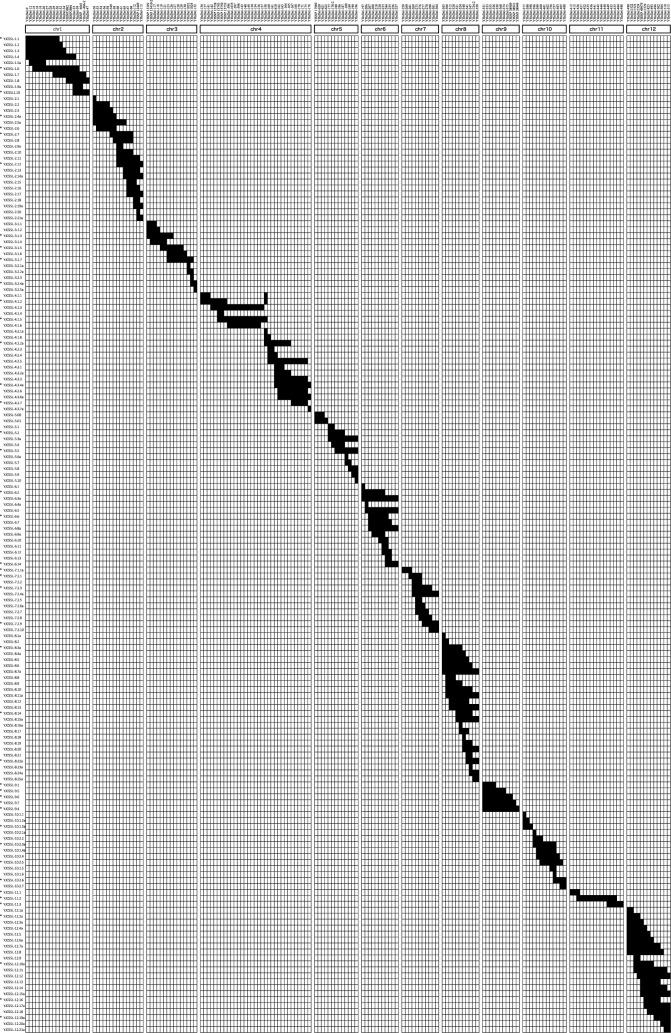
Graphical representation of the genotypes of the 167 chromosome segment substitution lines (CSSLs) generated in this study. White and black bars indicate homozygous chromosomal segments derived from ‘Joiku462’ and ‘Yukihikari’, respectively. The InDel and single nucleotide polymorphism (SNP), i.e., cleaved amplified polymorphic sequence (CAPS) and derived CAPS (dCAPS), markers used for marker-assisted selection (MAS) are indicated on each chromosome. Asterisks indicate the plant materials used in the field trial study.

All 167 ‘Yukihikari’, ‘Joiku462’ CSSLs (YJCSSLs) carried a single homozygous substituted chromosome segment, except for two CSSLs, i.e., YJCSSL-4.1.1 and YJCSSL-4.1.2, which carried two segments each (Fig. 2). Considering that recombination events occur at the midpoint between two adjacent markers, the target chromosomal segment ranged from 0.6 Mb on chromosome 5 to 29.7 Mb on chromosome 1, with a mean length of 10.7 Mb (Supplemental Table 1). Approximately 27% of substituted segments were smaller than 5 Mb, 26% of substituted segments ranged from 5 to 9.9 Mb, 17% ranged from 10 to 14.9 Mb, 15% ranged from 15 to 19.9 Mb, and 15% of substituted segments were over 20 Mb. The average coverage rate of the substitution segments per chromosome was 92.2%, ranging from 83.3% on chromosome 2 to 98.0% on chromosome 4. These CSSLs covered 92.1% (343.7 Mb) of the ‘Yukihikari’ genome. In a further experiment, 44 CSSLs that covered 92.1% of the ‘Yukihikari’ genome were evaluated for heading date and grain quality-related traits.

### Evaluation of DTH and grain quality-related traits

The DTH and grain quality-related traits of the parental cultivars and CSSLs under the two experimental field conditions are represented in Table 1. Six traits, including AAC, PC, TBGW, BGL, BGWI, and BGT, differed significantly between the parental cultivars in either one or both growth environments (*P* < 0.05). AAC and PC were lower in ‘Joiku462’ than in ‘Yukihikari’, whereas TBGW, BGL, BGWI, and BGT were higher in ‘Joiku462’ than in ‘Yukihikari. These results indicate that ‘Joiku462’ showed improved eating quality, with lower amylose and protein contents and better grain appearance, along with larger grains with DTH similar to that in ‘Yukihikari’, as reported in Kinoshita *et al.* (2017).

The concentrations of the 12 mineral elements in the polished rice of the parental cultivars and CSSLs are represented in Table 2. The contents of three of these elements, i.e., S, Zn, and Sr, differed significantly between the two parental cultivars in either one or both field environments (*P* < 0.05). The Zn content was lower in ‘Yukihikari’ than in ‘Joiku462’, whereas the S and Sr contents were higher in ‘Yukihikari’.

### QTL detection

In total, 143 significant changes were identified in the evaluated traits in the CSSLs compared with those of the recurrent parent (Table 1), and 71 QTLs were distributed across all 12 chromosomes (Table 3).

Eight QTLs were associated with DTH. Five QTLs, including *qDTHb2.1*, *qDTHb2.2*, *qDTHb3*, *qDTHb8.1*, and *qDTHb8.2*, exhibited the inhibitory effect of ‘Yukihikari’ alleles on DTH. The remaining three QTLs, *qDTHb6.1*, *qDTHb6.2*, and *qDTHb9*, showed the enhancement effect of ‘Yukihikari’ alleles on DTH. Five QTLs, *qDTHb2.1*, *qDTHb3*, *qDTHb6.1*, *qDTHb6.2*, and *qDTHb8.1*, were stable QTLs detected in both conditions, whereas three QTLs, *qDTHb2.2*, *qDTHb8.2*, and *qDTHb9* were unstable and were detected only in a single condition. Among these, three QTLs, *qDTHb3*, *qDTHb6.1*, and *qDTHb9*, were observed to be responsible for functional polymorphisms in the previously cloned genes based on our previous resequencing data (Takano *et al.* 2014) (Supplemental Table 2). In the *qDTHb3* region, the *OsFdC2*, *OsPhyC*, *Hd6*, *Hd16*, *OsCCT14*, and *OsMADS14* genes overlapped (Dai and Xue 2010, Doi *et al.* 2004, Li *et al.* 2015, Liu *et al.* 2016, Zhao *et al.* 2015). Among these, ‘Joiku462’ carried a premature stop codon identical to that in ‘Nipponbare’ at the *Hd6* locus, whereas ‘Yukihikari’ carried the wild-type allele. In *qDTHb6.1* region, five cloned genes, *Hd3a*, *Hd3b*, *OsHGW*, *Hd1*, and *OsSDG711*, were observed. Two neighboring genes, *Hd1* and *OsSDG711*, exhibited functional polymorphisms between ‘Yukihikari’ and ‘Joiku462’. In the *Hd1* coding region, G614A (R205Q) mutation was identified. In the *OsSDG711* coding region, G320T (C117F) and T1397C (V466A) mutations were identified. In the *qDTHb9* region, the A3029C (A1010D) mutation was identified in *OsTrx1* (Jiang *et al.* 2018).

Seven QTLs associated with AAC were identified. Six QTLs, including *qAACb1*, *qAACb2.1*, *qAACb2.2*, *qAACb3*, *qAACb7*, and *qAACb8*, exhibited the inhibitory effect of ‘Yukihikari’ alleles on AAC. Conversely, only *qAACb9* increased AAC under the effect of the associated ‘Yukihikari’ allele. Four QTLs, i.e., *qAACb1*, *qAACb2.1*, *qAACb3* and *qAACb9*, were stable, whereas three QTLs, i.e., *qAACb2.2*, *qAACb7*, and *qAACb8*, were detected in 2019. No functional mutations were identified in the cloned genes that overlapped with the QTLs identified in the current study.

Eight QTLs associated with PC, including *qPCb2.1*, *qPCb2.2*, *qPCb3*, *qPCb5*, *qPCb6*, *qPCb7*, *qPCb8*, and *qPCb10*, were detected and exhibited the enhancement effect of ‘Yukihikari’ alleles on PC. Of these, four QTLs, i.e., *qPCb2.1*, *qPCb3*, *qPCb7*, and *qPCb8*, were detected across the two field conditions, suggesting that they were stable, whereas the remaining four QTLs, i.e., *qPCb2.2*, *qPCb5*, *qPCb6*, and *qPCb10*, were only detected in 2020. In the *qPCb7* region, *RAG2* (Zhou *et al.* 2017) contained three amino acid substitutions, T14I, A67G, and W128G, between ‘Yukihikari’ and ‘Joiku462’.

In addition, eight QTLs associated with TBGW were identified. Three QTLs, i.e., *qTBGWb1*, *qTBGWb2*, and *qTBGWb8*, exhibited the enhancement effect of ‘Yukihikari’ alleles on TBGW. Five QTLs, i.e., *qTBGWb3*, *qTBGWb4*, *qTBGWb6*, *qTBGWb7*, and *qTBGWb9*, exhibited the enhancement effect of ‘Joiku462’ alleles on TBGW. Among these, four QTLs, i.e., *qTBGWb2*, *qTBGWb3*, *qTBGWb8*, and *qTBGWb9*, were stable QTLs detected in both 2019 and 2022, whereas the other four QTLs, i.e., *qTBGWb1*, *qTBGWb4*, *qTBGWb6*, and *qTBGWb7*, were detected only in 2019.

Nineteen QTLs associated with BGL were identified. Seven QTLs, including *qBGLb2*, *qBGLb3.2*, *qBGLb4.2*, *qBGLb5.2*, *qBGLb7.1*, *qBGLb8*, and *qBGLb11*, exhibited the enhancement effect of ‘Yukihikari’ alleles on BGL. Twelve QTLs, including *qBGLb1*, *qBGLb3.1*, *qBGLb3.3*, *qBGLb4.1*, *qBGLb4.3*, *qBGLb5.1*, *qBGLb6*, *qBGLb7.2*, *qBGLb9*, *qBGLb10.1*, *qBGLb10.2*, and *qBGLb12*, exhibited the enhancement effect of ‘Joiku462’ alleles on BGL. Nine QTLs, including *qBGLb1*, *qBGLb3.1*, *qBGLb4.3*, *qBGLb5.1*, *qBGLb6*, *qBGLb7.1*, *qBGLb7.2*, *qBGLb9*, and *qBGLb12*, were stable QTLs observed in both field trials, whereas 10 QTLs, including *qBGLb2*, *qBGLb3.2*, *qBGLb3.3*, *qBGLb4.1*, *qBGLb4.2*, *qBGLb5.2*, *qBGLb8*, *qBGLb10.1*, *qBGLb10.2*, and *qBGLb11*, were detected only in 2019.

Ten QTLs associated with BGWI were identified. Three QTLs, i.e., *qBGWIb1*, *qBGWIb2*, and *qBGWIb8*, exhibited the enhancement effect of ‘Yukihikari’ alleles on BGWI. Five QTLs, including *qBGWIb3*, *qBGWIb4*, *qBGWIb6*, *qBGWIb7*, and *qBGWIb9*, exhibited the enhancement effect of ‘Joiku462’ alleles on BGWI. Six QTLs, including *qBGWIb1*, *qBGWIb2*, *qBGWIb3.1*, *qBGWIb4.1*, *qBGWIb5*, and *qBGWIb10*, were stable QTLs detected in both the 2019 and 2020 field trials, whereas four QTLs, i.e., *qBGWIb3.2*, *qBGWIb6*, *qBGWIb8*, and *qBGWIb9*, were detected only in 2020.

In addition, 10 QTLs associated with BGT were identified. Four QTLs, i.e., *qBGTb1*, *qBGTb4.1*, *qBGTb8*, and *qBGTb10*, exhibited the enhancement effect of ‘Yukihikari’ alleles on BGT. Six QTLs, including *qBGTb3*, *qBGTb4.2*, *qBGTb6.1*, *qBGTb6.2*, *qBGTb7*, and *qBGTb9*, exhibited the enhancement effect of ‘Joiku462’ alleles on BGT. Four QTLs, i.e., *qBGTb1*, *qBGTb4.1*, *qBGTb7*, and *qBGTb10*, were stable QTLs detected in both 2019 and 2020, whereas six QTLs, including *qBGTb3*, *qBGTb4.2*, *qBGTb6.1*, *qBGTb6.2*, *qBGTb8*, and *qBGTb9*, were detected only in 2020.

In total, 92 cloned genes for grain size overlapped with the QTLs identified in this study (Supplemental Table 2). Among these, *DRW1* (Zhang *et al.* 2021) was reported in *qTBGW9*, *qBGL9*, and *qBGWI9* and exhibited a single amino acid substitution between the parental lines. No functional polymorphisms were identified in the other cloned genes.

Twenty-seven significant changes in S, Mo, Cu, and Zn contents were identified in the CSSLs compared with those in the recurrent parent, and eight QTLs were located on chromosomes 3, 6, 8, and 9 (Table 3). Two QTLs associated with S content, i.e., *qSb3* and *qSb8*, were detected across the two field conditions and exhibited the enhancement effect of ‘Yukihikari’ alleles on S content. A single QTL, *qMob3*, associated with Mo content was detected in 2020 and showed the enhancement effect of the ‘Yukihikari’ allele on Mo content. Three QTLs, *qCub3*, *qCub6*, and *qCub9*, associated with Cu content were detected across the two field conditions. *qCub3* showed the enhancement effect of the ‘Yukihikari’ allele on Cu content. Similarly, two QTLs, i.e., *qCub6* and *qCub9*, exhibited the enhancement effect of ‘Joiku462’ alleles on Cu content. Two QTLs, i.e., *qZnb8* and *qZnb9*, associated with Zn content were detected in both field conditions and exhibited the enhancement effect of ‘Joiku462’ alleles. *OsZIP4* (Os08g0207500) overlapped with *qZnb8* without revealing any functional polymorphisms (Supplemental Table 2). Although the Sr content in ‘Yukihikari’ was higher than that in ‘Joiku462’ across the 2 years of field trials, no significant differences in Sr content were detected between each CSSL and ‘Joiku462’. However, the Sr content in the CSSLs exhibited a positive correlation between the two trials (*r* = 0.70, *P* < 0.001). Across the 2 years of field trials, three CSSLs, YJCSSL-1.6, -9.5, and -9.6, exhibited higher Sr content than the other CSSLs, suggesting that these CSSLs carry small-effect QTLs that regulate Sr content.

## Discussion

### Newly identified QTLs in CSSL-QTL analysis

In this study, we identified 78 QTLs, including 8, 7, 8, 8, 19, 10, and 10 QTLs regulating DTH, AAC, PC, TBGW, BGL, BGWI, and BGT, respectively, and 2, 1, 3, and 2 QTLs regulating S, Mo, Cu, and Zn contents, respectively, in the genetic background of ‘Joiku462’ (superior eating and high grain appearance qualities). In our previous QTL analysis using the RIL population of the same cross combination, 36 QTLs, including 5, 3, 8, 7, 2, 4, and 7 QTLs regulating DTH, AAC, PC, TBGW, BGL, BGWI, and BGT, respectively, were reported (Kinoshita *et al.* 2017). Among them, 20 QTLs were located within the same or adjacent intervals in this study, indicating that these QTLs had stable effects on heading date and grain quality traits in the CSSLs and RILs. In contrast, 49 QTLs, including 5 (62.5%), 5 (71.4%), 4 (50.0%), 6 (75.0%), 18 (94.7%), 8 (80.0%), and 4 (40.0%) QTLs regulating DTH, AAC, PC, TBGW, BGL, BGWI, and BGT, respectively, were newly identified in the current study. These results indicate that CSSLs can help identify QTLs with relatively small genetic effects (Howell *et al.* 1996, Nagata *et al.* 2015, Okada *et al.* 2018).

### Co-localization of QTLs for grain quality-related traits with QTLs for DTH

The date of heading is a key agronomic trait in rice that determines the regional and seasonal adaptation of rice varieties and affects grain yield and quality. Only cultivars with an extremely early heading date were adapted to Hokkaido (41–45° N latitude), which has a long natural day length of more than 15 h in summer (Fujino and Sekiguchi 2005). Although similar DTH values were observed in the parental cultivars, the ‘Yukihikari’ cultivar possessed early heading date alleles at *qDTHb2.1*, *qDTHb2.2*, *qDTHb3*, *qDTHb8.1*, and *qDTHb8.2*, whereas ‘Joiku462’ possessed early heading date alleles at *qDTHb6.1*, *qDTHb6.2*, and *qDTHb9*.

Among the eight QTLs for DTH, four co-localized with the QTLs for AAC and PC and formed clusters *qDTHb2.1*–*qAACb2.1*–*qPCb2.1* (chr2: ~2.3 Mb), *qDTHb2.2*–*qAACb2.2*–*qPCb2.2* (chr2:15.8–35.9 Mb), *qDTHb3*–*qAACb3*–*qPCb3* (chr3:26.9–36.4 Mb), and *qDTHb8.1*–*qAACb8*–*qPCb8* (chr8:4.0–8.6 Mb). ‘Yukihikari’ alleles were associated with a consistent decrease in DTH and AAC and increased PC in these four clusters. Two QTLs, *qDTH3*–*qPC3* and *qDTH8*–*qAAC8*–*qPC8*, identified in our previous study using RIL population derived from a cross between ‘Yukihikari’ and ‘Joiku462’ (Kinoshita *et al.* 2017) overlapped with *qDTHb3*–*qAACb3*–*qPCb3* and *qDTHb8.1*–*qAACb8*–*qPCb8*, identified on chromosomes 3 and 8, respectively, in the current study. In agreement with the results obtained in the current study, *qDTH3*–*qPC3* and *qDTH8*–*qAAC8*–*qPC8* decrease DTH or AAC or both and increase PC under the effects of ‘Yukihikari’ alleles (Kinoshita *et al.* 2017). Therefore, these two QTL clusters had stable effects on DTH, AAC, and PC in CSSLs and RILs. Similarly, the co-localization of QTLs for DTH and PC has been reported earlier (Mo *et al.* 2021, Tan *et al.* 2001, Wada *et al.* 2006, Yun *et al.* 2016). Hitherto, the pleiotropic effects of the rice florigen gene *RFT1* on the amino acid content of rice grains have been reported (Xie *et al.* 2020). However, the pleiotropic effects of other genes determining the head date on PC have not yet been elucidated. Nevertheless, the effects of differences in ripening temperature because of differences in DTH must be considered (Kinoshita *et al.* 2017). Further studies are needed to determine whether DTH, AAC, and PC are controlled by closely linked QTLs or a pleiotropic QTL. In contrast, three QTLs, *qDTHb6.1*, *qDTHb6.2*, and *qDTHb9*, reduced DTH under the effects of ‘Joiku462’ alleles and did not co-localize with QTLs regulating AAC or PC. Among them, two QTLs, *qDTHb6.1* and *qDTHb6.2*, co-localized with QTLs for grain size and formed the clusters *qDTHb6.1*–*qTBGWb6*–*qBGLb6* and *qDTHb6.2*–*qBGWIb6*–*qBGTb6.2*. Both QTL clusters decreased DTH and increased grain size under the effects of the ‘Joiku462’ alleles.

Taken together, the ‘Yukihikari’ cultivar carrying five QTLs, including *qDTHb2.1*, *qDTHb2.2*, *qDTHb3*, *qDTHb8.1*, and *qDTHb8.2*, exhibited an extremely early heading date. These five QTLs can be classified into two categories: the first category consists of *qDTHb2.1*, *qDTHb2.2*, *qDTHb3*, and *qDTHb8.1*, which co-localized with QTLs that reduce AAC and enhance PC, and the second category consists of *qDTHb8.2*, which did not co-localize with QTLs regulating other traits. Similarly, the ‘Joiku462’ cultivar carrying three QTLs, i.e., *qDTHb6.1*, *qDTHb6.2*, and *qDTHb9*, exhibited an extremely early heading date. These three QTLs can be classified into two categories: the first category consists of *qDTHb6.1* and *qDTHb6.2*, which co-localized with QTLs promoting adequate grain shape, and the second category consists of *qDTHb9*, which did not exhibit co-localization with QTLs regulating the other traits.

In addition, the *qDTHb3*–*qAACb3*–*qPCb3* cluster co-localized with additional seven QTLs, including *qTBGWb3*, *qBGLb3.3*, *qBGWIb3.2*, *qBGTb3*, *qSb3*, *qMob3*, and *qCub3*, and formed the largest cluster in the region of 26.9–36.4 Mb on chromosome 3. In this cluster, the ‘Yukihikari’ allele was associated with decreased DTH, AAC, and grain size (TBGW, BGL, BGWI, and BGT) and with increased PC and mineral contents (S, Mo, and Cu). Fourteen cloned genes were identified within this cluster (Supplemental Table 2). A functional mutation at *Hd6* (OsCKA2) within this cluster was detected between ‘Yukihikari’ and ‘Joiku462’. Although the ‘Joiku462’ cultivar carries a premature stop codon at *Hd6*, the ‘Joiku462’ allele at *qDTHb3*/*qDTH3* delayed heading compared with that caused by the wild-type allele of ‘Yukihikari’ (Kinoshita *et al.* 2017, Takano *et al.* 2014, and the current study). Takahashi *et al.* (2001) reported that ‘Nipponbare’ carries a premature stop codon at *Hd6* and causes early heading compared with that caused by the wild-type allele of ‘Kasalath’. Further studies are needed to resolve this conflict and investigate two possibilities: first, the variation in the expression of five cloned genes, including *OsFdC2*, *OsPhyC*, *Hd16*, *OsCCT14*, and *OsMADS14* (Dai and Xue 2010, Doi *et al.* 2004, Li *et al.* 2015, Liu *et al.* 2016, Zhao *et al.* 2015), regulates DTH; and second, a novel gene controlling DTH is included in *qDTHb3*/*qDTH3*.

CK2 is a heterotetramer consisting of two catalytic subunits (A or A′ or both) and two regulatory subunits (B). CK2 can affect DNA binding by phosphorylating a range of transcription factors, which, in turn, affect the expression of downstream genes. In maize, CK2 regulates seed development by modulating the phosphorylation status of the bZIP transcriptional activator Opaque2, which is specifically expressed during endosperm development and enhances the transcriptional synthesis of seed storage proteins (Ciceri *et al.* 1997). Future studies will be needed to determine whether a non-functional allele at *OsCKA2* derived from ‘Joiku462’ reduces protein accumulation or whether another gene controls protein accumulation in rice.

### QTLs regulating AAC and PC that are independent of QTLs regulating DTH

*qAACb9* co-localized with five QTLs, including *qTBGWb9*, *qBGLb9*, *qBGWIb9*, *qBGTb9*, and *qZnb9*, forming a second large QTL cluster. In this cluster, the ‘Joiku462’ allele was associated with reduced AAC and increased grain size and Zn content. *qAACb9* was identified at a position identical to that of *qAC9/qAAC9* using RILs derived from the crosses of ‘Joiku462’ with ‘Jokei06214’ and ‘Yukihikari’ with ‘Joiku462’, respectively (Kinoshita *et al.* 2017, Shinada *et al.* 2015). This result indicates that the allele at *qAAC9/qAC9*/*qAACb9* derived from ‘Joiku462’ exhibits a stable reduction effect on AAC in different genetic backgrounds and environmental conditions. In addition, QTLs for grain shape-related traits, i.e., *qTBGW9*, *qBGWI*, and *qBGT9*, were shown to co-localize with *qAAC9* in our previous study using RILs derived from the same cross combination (Kinoshita *et al.* 2017). *qAAC9/qAC9*/*qAACb9*, *qAC-9-1*(t), and *qAC9* have been detected in different cross combinations (Wada *et al.* 2006, Wan *et al.* 2004). Further studies are required to determine the precise positions of QTLs for multiple traits in QTL clusters and to assess whether pleiotropic effects are because of single or closely linked QTLs.

All eight QTLs for PC exhibited an increase in PC caused by the ‘Yukihikari’ allele in the genetic background of ‘Joiku462’ (superior eating quality). Among these, four QTLs, i.e., *qPCb5*, *qPCb6*, *qPCb7*, and *qPCb10*, were independent of the QTLs determining DTH. *RAG2* located within *qPCb7* and contained three functional mutations, T14I, A67G, and W128G, between ‘Yukihikari’ and ‘Joiku462’ based on our previous resequencing data (Takano *et al.* 2014). The seeds of RAG2-overexpressing and RAG2-suppressed lines exhibited increased and decreased PC, respectively (Zhou *et al.* 2017). Further studies are required to determine whether natural variations in *RAG2* regulate PC.

### QTL clusters for grain shape-related traits

‘Joiku462’ exhibited improved grain appearance, involving larger BGL, BGWI, or BGT than in ‘Yukihikari’. ‘Joiku462’ yielded consistently stable thick brown rice grains of thickness >2.0 mm, whereas ‘Yukihikari’ yielded grains of thickness <2.0 mm in the current study. In the current study, we identified 47 QTLs determining grain size distributed across all chromosomes. Among these, 30 QTLs (63.8%) were identified in common for TBGW, BGL, BGWI, and BGT and formed 11 clusters involving *qTBGWb3*–*qBGLb3.3*–*qBGWIb3.2*–*qBGTb3* (chr3:26.9–36.4 Mb), *qTBGWb6*–*qBGLb6* (chr6:2.0–21.2 Mb), *qBGWIb6*–*qBGTb6.2* (chr6:21.2–31.2 Mb), *qTBGWb1*–*qBGWIb1*–*qBGTb1* (chr1:34.4–43.3 Mb), *qTBGWb2*–*qBGLb2*–*qBGWIb2* (chr2: ~2.3 Mb), *qBGLb4.1*–*qBGWIb4.1* (chr4: ~8.1 Mb), *qBGLb5.1*–*qBGWIb5* (chr5:1.1–13.5 Mb), *qTBGWb7*–*qBGLb7.2*–*qBGTb7* (chr7:8.7–19.0 Mb), *qTBGWb8*–*qBGLb8*–*qBGTb8* (chr8: ~5.1 Mb), *qTBGWb9*–*qBGLb9*–*qBGWIb9*–*qBGTb9* (chr9: ~8.8 Mb), and *qBGWIb10*–*qBGTb10* (chr10: ~12.7 Mb). These clusters were classified into three groups: the first group consisted of six clusters, including *qTBGWb3*–*qBGLb3.3*–*qBGWIb3.2*–*qBGTb3*, *qTBGWb6*–*qBGLb6*, *qBGWIb6*–*qBGTb6.2*, *qTBGWb7*–*qBGLb7.2*–*qBGTb7*, *qTBGWb9*–*qBGLb9*–*qBGWIb9*–*qBGTb9*, and *qBGWIb10*–*qBGTb10*, increased grain size and was associated with the ‘Joiku462’ allele; the second group consisting of three clusters, i.e., *qTBGWb1*–*qBGWIb1*–*qBGTb1*, *qTBGWb2*–*qBGLb2*–*qBGWIb2*, and *qTBGWb8*–*qBGLb8*–*qBGTb8*, increased grain size and was associated with the ‘Yukihikari’ allele; and the third group consisting of two clusters, i.e., *qBGLb4.1*–*qBGWIb4.1* and *qBGLb5.1*–*qBGWIb5*, increased BGL together with reduced BGWI, resulting in slender grains and was associated with the ‘Joiku462’ allele.

The present 60 QTLs overlapped with 164 cloned genes, including 23, 11, 10, 33, 68, 14, 7, and 1, determining DTH, AAC, PC, TBGW, BGL, BGWI, BGT, and Zn, respectively. Among them, seven cloned genes (five for DTH, one for PC, and one each for TBGW, BGL, and BGWI) contained functional mutations. Of the currently known eight QTLs for mineral content, only *qZnb8* overlapped *OsZIP4* (Os08g0207500) without any functional mutation (Takano *et al.* 2014). Sixty of the 78 QTLs (76.9%) co-localized with other QTLs and formed 17 clusters on chromosomes 1–10. These QTL cluster regions are responsible for genetic features, such as linkage drag and pleiotropy, and have critical implications in rice breeding. These QTLs can facilitate gene isolation and breeding to develop rice cultivars with optimum heading time and improved grain quality.

## Author Contribution Statement

KK, SM, TS, and TW designed the study and performed experiments. YH analyzed the data. KK, SM, and TW wrote the manuscript. All the authors have read and approved the final version of the manuscript.

## Supplementary Material

Supplemental Table 1

Supplemental Table 2

## Figures and Tables

**Table 1.** Average phenotypic values of parents and a comparative analysis of each chromosome segment substitution line (CSSL) with ‘Joiku462’

Genotype	DTH					AAC (%)					PC (%)					TBGW (g)					BGL (mm)					BGWI (mm)					BGT (mm)			
	2019P		2020P			2019P		2020P			2019P		2020P			2019P		2020P			2019P		2020P			2019P		2020P			2019P		2020P	
Joiku462	95.3		87.0			20.6		19.4			5.6		5.4			25.4		26.7			5.11		5.46			2.98		2.84			2.02		2.03	
Yukihikari	96.3		87.0			22.8	***	20.8	**		6.3	**	7.0	***		23.0	***	23.6	***		4.86	***	5.19	***		2.92		2.79	***		1.99	**	1.97	***
YJCSSL-1.1	94.7		85.3			19.5		18.1	*		5.8		5.5			25.0		26.4			4.99	***	5.33	***		2.96		2.85			2.03		2.05	
YJCSSL-1.6	95.3		87.0			19.3	*	18.3			5.5		5.4			25.4		26.3			5.13		5.45			3.00		2.85			2.03		2.01	
YJCSSL-1.10	95.7		86.3			20.9		19.5			5.8		5.6			26.6	*	27.4			5.13		5.43			3.06	**	2.87	*		2.05	*	2.07	***
YJCSSL-2.4a	91.3	**	82.0	***		18.7	***	17.6	***		6.5	**	6.2	***		25.9		26.7			5.13		5.45			2.97		2.84			2.02		2.04	
YJCSSL-2.6	94.3		86.3			19.5		18.3			5.6		5.4			25.3		26.4			5.18	***	5.46			2.99		2.85			2.03		2.02	
YJCSSL-2.7	93.3		83.3	**		19.3	*	18.3			6.0		6.2	***		25.3		26.7			5.09		5.41			3.00		2.85			2.03		2.04	
YJCSSL-2.12	95.3		86.7			20.7		19.4			6.0		5.9			27.0	***	28.6	***		5.17	***	5.52			3.07	**	2.88	***		2.05	.	2.05	
YJCSSL-3.1.3	95.7		87.0			21.2		19.8			5.9		5.7			26.1		27.2			5.12		5.44			3.03	*	2.86			2.04		2.03	
YJCSSL-3.1.5	95.3		86.7			20.3		18.8			5.6		5.8			25.0		26.5			5.04	**	5.32	***		2.99		2.84			2.03		2.04	
YJCSSL-3.1.7	94.0		85.0			21.0		19.5			5.7		5.8			26.3		26.8			5.20	***	5.49			2.97		2.83			2.03		2.02	
YJCSSL-3.2.4a	91.7	**	83.3	**		18.7	***	17.5	***		7.2	**	7.3	***		23.3	***	25.1	**		4.99	***	5.38			2.89		2.81	***		2.00		1.99	***
YJCSSL-4.1.2	93.3		85.3			20.8		19.7			5.8		5.7			26.1		27.6			5.05	*	5.40			3.04	*	2.87	**		2.05	**	2.08	***
YJCSSL-4.1.5	95.0		86.3			21.2		20.2			5.8		5.7			25.7		27.6			5.17	**	5.48			2.98		2.85			2.04		2.06	*
YJCSSL-4.2.2a	95.3		87.0			20.8		19.0			5.8		5.8			24.0	**	26.0			4.99	***	5.29	***		2.96		2.83			2.02		2.03	
YJCSSL-4.3.4a	94.3		84.3	.		20.5		19.1			6.0		5.8			25.2		26.4			5.04	**	5.35	***		2.98		2.84			2.03		2.04	
YJCSSL-4.3.7a	95.3		87.0			20.8		19.2			5.8		5.8			25.5		26.9			5.13		5.48			3.02	.	2.84			2.02		2.01	*
YJCSSL-5.2	94.3		88.3			20.8		19.7			6.0		5.9	*		25.1		27.0			5.04	**	5.37	*		3.01		2.87	*		2.03		2.04	
YJCSSL-5.5	94.3		87.7			20.5		19.2			6.0		5.8			26.0		27.8			5.17	***	5.52			3.03	*	2.87	**		2.04		2.04	
YJCSSL-6.2	99.3	**	92.3	***		19.9		19.4			5.8		5.8	*		23.7	***	25.9			5.00	***	5.45	***		2.99		2.79			2.01		1.99	*
YJCSSL-6.6	94.3		91.3	***		20.0		18.8			5.8		5.9			24.1	**	25.9			5.01	***	5.31	**		2.97		2.82			2.02		2.01	
YJCSSL-6.14	100.3	***	86.3			20.0		19.3			5.4		5.9			24.2	*	26.9			5.06	.	5.35			2.90		2.83	***		2.00	.	2.02	***
YJCSSL-7.1.1a	94.3		86.0			20.0		19.3			5.8		5.7			25.7		27.5			5.14		5.50			2.99		2.84			2.03		2.03	
YJCSSL-7.2.1	94.7		86.7			20.5		19.6			6.1		6.0	*		25.7		28.0			5.18	***	5.56	**		2.99		2.84			2.03		2.02	
YJCSSL-7.2.3	95.7		86.7			19.9		19.1			6.4	**	6.1	**		23.3	***	26.1			4.98	***	5.34	***		2.95		2.83			1.99	**	1.98	***
YJCSSL-7.2.9	94.3		85.7			19.4	*	18.8			5.8		5.9			25.2		26.7			5.11		5.45			2.98		2.84			2.02		2.03	
YJCSSL-8.3a	85.0	***	78.7	***		18.9	***	18.3			6.8	**	6.5	***		26.6	*	28.0	*		5.23	***	5.51			2.97		2.85			2.05		2.07	**
YJCSSL-8.14	84.7	***	80.3	***		19.1	**	18.5			7.0	**	6.7	***		23.9	**	26.8			5.09		5.44			2.85		2.82	*		2.01		2.02	
YJCSSL-8.22a	92.3	*	87.0			20.5		19.7			5.8		5.7			25.0		27.8			5.13		5.51			2.95		2.84			2.03		2.03	
YJCSSL-9.1	94.7		90.0	*		23.2	***	21.8	***		5.8		5.7			23.9	**	25.2	*		5.01	***	5.38	*		2.94		2.82	*		2.01		1.99	***
YJCSSL-9.5	95.3		89.0			23.7	***	22.4	***		5.5		5.8			23.6	***	24.9	***		4.94	***	5.25	***		2.97		2.83			2.02		2.01	*
YJCSSL-9.6	93.7		89.7			24.0	***	22.7	***		5.5		5.6			24.7		25.2	*		5.07		5.39			2.97		2.82	*		2.02		1.99	***
YJCSSL-9.7	94.3		88.3			24.3	***	22.7	***		5.6		5.5			24.2	*	25.6			5.07		5.43			2.97		2.82	*		2.02		2.00	**
YJCSSL-9.4	94.0		87.7			24.4	***	22.7	***		5.7		5.7			25.1		25.8			5.11		5.47			2.97		2.83			2.01		2.00	***
YJCSSL-10.1.3a	94.3		89.3			21.0		20.3			5.5		5.6			25.9		27.5			5.07		5.44			3.03	*	2.87	**		2.05	*	2.06	*
YJCSSL-10.2.3a	93.3		87.0			19.9		19.4			5.9		5.9	*		25.1		27.1			5.04	***	5.46			2.99		2.86			2.03		2.03	
YJCSSL-10.2.5	94.3		87.0			21.2		20.2			5.6		5.4			24.9		27.5			5.07		5.45			2.99		2.85			2.03		2.04	
YJCSSL-10.2.6	93.3		85.0			20.2		19.6			5.6		5.6			25.3		26.8			5.04	**	5.43			3.00		2.84			2.04		2.04	
YJCSSL-11.1	95.0		87.3			21.0		19.6			5.4		5.5			25.3		26.5			5.16	**	5.47			2.96		2.83			2.02		2.02	
YJCSSL-11.2	93.0		84.7			21.2		19.9			5.7		5.6			25.4		27.0			5.12		5.48			2.96		2.83			2.03		2.02	
YJCSSL-11.3	94.7		86.3			20.6		19.4			5.5		5.6			25.0		26.3			5.11		5.46			3.00		2.83			2.03		2.03	
YJCSSL-12.2a	94.7		88.3			20.6		19.4			5.6		5.5			25.1		26.2			5.06		5.37			3.00		2.83			2.02		2.02	
YJCSSL-12.10a	93.7		85.7			20.7		19.2			5.6		5.5			24.8		26.7			5.07		5.41			3.00		2.84			2.02		2.03	
YJCSSL-12.16	94.3		86.3			20.8		18.8			5.8		5.8			24.3	.	26.7			5.04	**	5.38	***		3.00		2.86			2.03		2.04	
YJCSSL-12.19a	94.0		86.0			20.6		19.2			5.5		5.7			25.1		25.8			5.02	***	5.30	*		2.99		2.83			2.02		2.03	

Dunnett’s multiple comparison test was conducted for each trait to compare Joiku462 with each CSSL, and “*”, “**” and “***” represented significant at *P* < 0.05, *P* < 0.01 and *P* < 0.001, respectively.

**Table 2.** Average 12 elements in the polished rice obtained from parents and a comparative analysis of each chromosome segment substitution line (CSSL) with ‘Joiku462’

Genotype	S (mg/g)					P (mg/g)					Mg (mg/g)					Ca (mg/g)					K (mg/g)					Mo (μg/g)					Cu (μg/g)					Zn (μg/g)					Mn (μg/g)					Fe (μg/g)					Sr (μg/g)					Acid soluble Si (μg/g)			
	2019P		2020P			2019P		2020P			2019P		2020P			2019P		2020P			2019P		2020P			2019P		2020P			2019P		2020P			2019P		2020P			2019P		2020P			2019P		2020P			2019P		2020P			2019P		2020P	
Joiku462	0.72		0.66			0.87		1.13			0.17		0.35			0.090		0.115			0.63		1.00			1.25		1.01			2.89		2.77			15.6		14.6			10.1		13.1			1.55		1.93			0.046		0.061			30.5		40.5	
Yukihikari	0.75		0.79	**		0.99		1.39			0.20		0.42	.		0.104		0.132			0.65		1.11			1.42		1.20			2.48		2.43			11.4	***	11.4	***		10.6		13.1			2.29		2.77			0.063	**	0.081	*		29.6		32.2	
YJCSSL-1.1	0.71		0.70			0.84		1.11			0.17		0.32			0.088		0.111			0.59		0.96			1.32		1.09			2.81		2.85			16.2		16.2			9.9		12.1			1.79		1.64			0.046		0.055			27.5		35.0	
YJCSSL-1.6	0.72		0.68			0.88		1.15			0.18		0.36			0.088		0.124			0.62		1.02			1.38		1.12			2.76		2.86			15.5		13.5			10.3		14.0			1.66		2.14			0.057		0.067			35.3		43.3	
YJCSSL-1.10	0.71		0.69			0.83		1.12			0.17		0.36			0.080		0.105			0.59		1.01			1.18		1.12			2.62		2.80			15.5		15.2			9.8		12.8			1.96		1.76			0.042		0.057			22.7		34.3	
YJCSSL-2.4a	0.78		0.77	.		0.88		1.22			0.19		0.38			0.087		0.119			0.57		1.03			1.30		1.26	.		2.83		2.99			14.9		14.6			10.2		13.5			1.86		1.92			0.045		0.064			27.2		38.8	
YJCSSL-2.6	0.70		0.69			0.84		1.15			0.18		0.34			0.089		0.122			0.61		0.97			1.13		1.12			2.74		2.92			14.3		13.7			10.7		14.0			ND		1.54			0.050		0.063			30.4		40.8	
YJCSSL-2.7	0.75		0.74			0.85		1.14			0.18		0.34			0.090		0.116			0.59		0.89			1.15		1.14			2.91		2.97			15.3		14.2			10.5		12.8			1.59		1.62			0.047		0.060			32.5		40.3	
YJCSSL-2.12	0.74		0.72			0.84		1.08			0.17		0.31			0.088		0.120			0.55		0.85			1.13		1.13			2.74		2.79			15.3		14.1			10.5		13.4			1.47		1.64			0.048		0.062			38.8		45.1	
YJCSSL-3.1.3	0.71		0.69			0.83		1.17			0.17		0.33			0.085		0.113			0.58		0.91			1.07		1.04			2.78		2.77			15.2		14.6			10.1		12.7			1.56		2.94			0.042		0.056			35.6		38.2	
YJCSSL-3.1.5	0.68		0.70			0.76		1.13			0.16		0.33			0.083		0.117			0.57		0.91			1.01		1.05			2.70		3.07			15.2		15.2			9.9		13.1			1.36		1.65			0.041		0.055			29.4		45.0	
YJCSSL-3.1.7	0.69		0.71			0.73		1.06			0.15		0.33			0.079		0.111			0.51		0.86			1.05		1.06			2.74		2.96			15.1		15.3			9.0		12.2			1.78		1.76			0.038		0.053			31.7		35.2	
YJCSSL-3.2.4a	0.86	***	0.85	***		0.95		1.30			0.23	.	0.40			0.088		0.114			0.61		0.99			1.28		1.28	*		3.42	*	3.37	*		17.0		15.6			11.4		13.5			1.81		2.72			0.046		0.059			32.0		34.9	
YJCSSL-4.1.2	0.71		0.69			0.80		1.03			0.18		0.31			0.084		0.112			0.59		0.82			1.06		1.00			2.85		2.92			14.8		14.4			10.4		12.7			1.64		1.89			0.044		0.055			28.7		36.3	
YJCSSL-4.1.5	0.71		0.68			0.75		1.06			0.16		0.31			0.080		0.110			0.59		0.89			1.04		0.98			2.58		2.76			14.1		14.3			10.1		12.9			1.50		1.90			0.042		0.056			24.1		37.5	
YJCSSL-4.2.2a	0.70		0.69			0.80		1.11			0.17		0.36			0.085		0.117			0.59		0.88			1.05		1.00			2.71		2.89			15.0		14.6			10.0		13.1			1.65		1.87			0.044		0.058			29.5		40.3	
YJCSSL-4.3.4a	0.73		0.68			0.80		1.01			0.18		0.32			0.084		0.107			0.58		0.87			1.25		1.03			2.52		2.59			14.5		13.1			9.0		11.3			1.57		1.78			0.043		0.056			32.1		33.6	
YJCSSL-4.3.7a	0.73		0.67			0.79		1.05			0.16		0.33			0.083		0.112			0.56		0.88			1.13		0.98			2.82		2.65			15.3		13.7			10.4		13.1			1.48		2.04			0.043		0.056			30.6		37.3	
YJCSSL-5.2	0.72		0.70			0.79		1.09			0.16		0.34			0.081		0.106			0.56		0.87			1.10		1.05			2.70		3.01			15.4		14.9			10.0		12.9			1.46		1.79			0.043		0.053			33.2		36.0	
YJCSSL-5.5	0.73		0.69			0.77		1.04			0.16		0.33			0.081		0.105			0.56		0.83			1.19		1.10			2.86		2.83			15.4		14.7			10.2		12.5			1.61		2.61			0.042		0.051			35.5		35.6	
YJCSSL-6.2	0.72		0.71			0.84		1.04			0.17		0.31			0.087		0.105			0.68		0.91			1.00		1.03			2.29	*	2.28	*		13.7	.	13.8			10.0		11.8			1.64		2.35			0.048		0.057			36.8		32.1	
YJCSSL-6.6	0.72		0.70			0.82		1.12			0.19		0.38			0.090		0.114			0.62		0.94			1.14		1.04			2.59		2.85			16.1		15.6			10.0		12.7			1.68		2.06			0.046		0.057			31.7		38.6	
YJCSSL-6.14	0.72		0.73			0.78		1.12			0.17		0.38			0.084		0.113			0.56		0.92			1.24		1.10			2.78		2.93			15.9		16.1			9.8		13.6			1.69		2.97			0.044		0.058			30.2		32.3	
YJCSSL-7.1.1a	0.72		0.69			0.80		1.07			0.17		0.35			0.088		0.112			0.57		0.87			1.23		1.08			2.71		2.71			14.2		13.8			10.0		12.9			1.43		5.42			0.043		0.056			34.4		34.8	
YJCSSL-7.2.1	0.77		0.72			0.76		1.06			0.17		0.32			0.081		0.110			0.58		0.88			1.41		1.11			2.71		2.76			14.8		14.4			10.5		13.1			1.56		1.97			0.044		0.055			25.3		37.6	
YJCSSL-7.2.3	0.80		0.74			0.79		1.11			0.19		0.36			0.081		0.122			0.59		0.90			1.57		1.18			2.89		2.78			15.6		15.4			9.7		12.5			1.83		3.35			0.045		0.065			27.1		37.4	
YJCSSL-7.2.9	0.72		0.72			0.77		1.05			0.17		0.34			0.084		0.111			0.58		0.91			1.28		1.08			2.80		3.07			14.8		14.8			10.0		13.1			1.62		2.16			0.045		0.058			27.6		33.3	
YJCSSL-8.3a	0.83	**	0.81	***		0.85		1.12			0.18		0.32			0.080		0.105			0.56		0.84			1.45		1.25	.		2.78		2.92			13.5	*	13.8			9.3		11.4			1.61		2.46			0.041		0.053			27.9		31.4	
YJCSSL-8.14	0.85	***	0.79	*		0.89		1.10			0.19		0.32			0.084		0.103			0.58		0.88			1.34		1.23			2.95		2.79			13.5	*	13.1			10.2		11.5			1.57		2.30			0.043		0.054			37.2		32.3	
YJCSSL-8.22a	0.72		0.69			0.86		1.03			0.18		0.31			0.089		0.110			0.60		0.82			1.04		1.10			2.60		2.72			14.2		13.6			10.0		12.3			1.63		1.89			0.047		0.055			38.0		41.8	
YJCSSL-9.1	0.70		0.65			0.85		1.11			0.18		0.34			0.090		0.116			0.67		0.99			0.94		1.02			2.41		2.66			13.2	**	12.6			10.6		13.6			1.64		2.06			0.049		0.063			27.2		42.6	
YJCSSL-9.5	0.71		0.66			0.86		1.20			0.17		0.36			0.088		0.115			0.65		1.02			1.01		0.97			2.39		2.53			12.5	***	12.0	**		10.0		13.4			1.41		1.96			0.050		0.070			30.0		33.9	
YJCSSL-9.6	0.71		0.63			0.83		1.14			0.18		0.35			0.088		0.116			0.64		0.97			1.00		0.91			2.35	*	2.45	*		12.2	***	11.7	**		10.2		13.2			1.47		2.34			0.052		0.068			28.3		37.4	
YJCSSL-9.7	0.72		0.64			0.84		1.15			0.17		0.31			0.084		0.114			0.67		0.96			0.98		0.94			2.36	*	2.50	*		12.2	***	11.7	**		9.5		12.1			1.46		2.35			0.049		0.065			32.2		33.6	
YJCSSL-9.4	0.72		0.67			0.82		1.31			0.18		0.36			0.086		0.118			0.64		1.10			1.04		0.98			2.36	*	2.46	*		12.7	***	12.8			9.8		12.5			1.49		3.24			0.048		0.065			29.4		35.7	
YJCSSL-10.1.3a	0.70		0.67			0.75		1.17			0.15		0.33			0.077		0.118			0.57		0.92			1.01		1.01			2.58		3.05			15.9		15.8			9.7		13.6			1.30		2.15			0.040		0.058			26.8		43.2	
YJCSSL-10.2.3a	0.75		0.70			0.85		1.10			0.18		0.32			0.081		0.112			0.60		0.88			1.03		1.05			2.91		2.94			14.5		14.0			10.8		13.7			1.40		1.87			0.043		0.059			32.8		33.0	
YJCSSL-10.2.5	0.71		0.67			0.75		1.02			0.15		0.31			0.080		0.117			0.53		0.81			1.00		1.06			2.58		2.79			13.9		14.6			10.2		13.0			1.31		2.75			0.040		0.057			30.5		39.8	
YJCSSL-10.2.6	0.71		0.67			0.77		1.02			0.16		0.31			0.077		0.111			0.55		0.83			1.05		1.07			2.65		2.74			14.0		13.8			9.7		12.3			1.57		1.25			0.042		0.057			31.8		38.5	
YJCSSL-11.1	0.68		0.65			0.76		1.06			0.17		0.33			0.082		0.115			0.59		0.87			0.96		1.00			2.58		2.70			14.2		13.7			10.4		13.3			1.42		1.39			0.044		0.061			26.9		36.4	
YJCSSL-11.2	0.72		0.67			0.78		1.04			0.16		0.29			0.076		0.112			0.58		0.82			1.05		1.04			2.64		2.82			14.3		13.9			9.9		12.7			1.38		1.12			0.039		0.056			29.9		40.0	
YJCSSL-11.3	0.72		0.67			0.79		1.06			0.16		0.31			0.083		0.113			0.60		0.87			1.15		1.05			2.61		2.77			14.0		13.3			10.0		12.7			1.39		1.67			0.044		0.060			33.6		38.9	
YJCSSL-12.2a	0.70		0.66			0.79		1.05			0.17		0.30			0.083		0.112			0.58		0.85			1.10		1.05			2.63		2.81			14.7		14.5			10.2		12.5			1.35		1.45			0.044		0.058			31.5		36.2	
YJCSSL-12.10a	0.72		0.69			0.77		1.11			0.16		0.30			0.078		0.109			0.57		0.91			1.09		1.11			2.67		2.78			15.0		13.7			10.0		12.5			1.45		1.80			0.040		0.057			30.7		36.6	
YJCSSL-12.16	0.69		0.67			0.76		1.05			0.17		0.30			0.080		0.111			0.57		0.81			1.09		1.12			2.74		3.54			16.4		16.0			10.4		12.7			1.35		1.46			0.044		0.058			27.5		39.7	
YJCSSL-12.19a	0.70		0.65			0.80		1.05			0.18		0.31			0.086		0.120			0.57		0.82			1.10		1.02			2.62		2.81			15.3		14.2			9.9		13.2			1.59		1.80			0.045		0.068			31.8		35.9	

Dunnett’s multiple comparison test was conducted for each trait to compare Joiku462 with each CSSL (n = 5), and “*”, “**” and “***” represented significant at *P* < 0.05, *P* < 0.01 and *P* < 0.001, respectively.

**Table 3.** Quantitative trait loci (QTLs) for days to heading and grain-quality-related traits

Trait	QTL	Chr	Position (IRGSP-1)		Representative CSSL	Trial	Positive allele
			start	end			
DTH	*qDTHb2.1*	2	1	2,337,026	YJCSSL-2.4a	19P, 20P	J
	*qDTHb2.2*	2	4,790,694	7,976,120	YJCSSL-2.7	20P	J
	*qDTHb3*	3	26,908,494	36,413,819	YJCSSL-3.2.4a	19P, 20P	J
	*qDTHb6.1*	6	2,002,736	21,248,529	YJCSSL-6.2, YJCSSL-6.6	19P, 20P	Y
	*qDTHb6.2*	6	21,248,529	31,248,787	YJCSSL-6.14	19P, 20P	Y
	*qDTHb8.1*	8	4,063,287	16,939,665	YJCSSL-8.3a, YJCSSL-8.14	19P, 20P	J
	*qDTHb8.2*	8	18,348,247	28,443,022	YJCSSL-8.22a	19P	J
	*qDTHb9*	9	1	3,057,249	YJCSSL-9.1	20P	Y
AAC	*qAACb1*	1	8,710,907	24,347,903	YJCSSL-1.1, YJCSSL-1.6	19P, 20P	J
	*qAACb2.1*	2	1	2,337,026	YJCSSL-2.4a	19P, 20P	J
	*qAACb2.2*	2	4,790,694	7,976,120	YJCSSL-2.7	19P	J
	*qAACb3*	3	26,908,494	36,413,819	YJCSSL-3.2.4a	19P, 20P	J
	*qAACb7*	7	23,158,649	29,697,621	YJCSSL-7.2.9	19P	J
	*qAACb8*	8	4,063,287	8,643,075	YJCSSL-8.3a, YJCSSL-8.14	19P	J
	*qAACb9*	9	1	8,781,870	YJCSSL-9.1, YJCSSL-9.5, YJCSSL-9.6, YJCSSL-9.7, YJCSSL-9.4	19P, 20P	Y
PC	*qPCb2.1*	2	1	2,337,026	YJCSSL-2.4a	19P, 20P	Y
	*qPCb2.2*	2	4,790,694	7,976,120	YJCSSL-2.7	20P	Y
	*qPCb3*	3	26,908,494	36,413,819	YJCSSL-3.2.4a	19P, 20P	Y
	*qPCb5*	5	1,111,445	13,459,969	YJCSSL-5.2	20P	Y
	*qPCb6*	6	1	5,155,721	YJCSSL-6.2	20P	Y
	*qPCb7*	7	3,359,562	18,964,912	YJCSSL-7.2.1, YJCSSL-7.2.3	19P, 20P	Y
	*qPCb8*	8	4,063,287	8,643,075	YJCSSL-8.3a, YJCSSL-8.14	19P, 20P	Y
	*qPCb10*	10	10,761,946	16,460,614	YJCSSL-10.2.3a	20P	Y
TBGW	*qTBGWb1*	1	34,385,285	43,270,923	YJCSSL-1.10	19P	Y
	*qTBGWb2*	2	15,833,180	35,937,250	YJCSSL-2.12	19P, 20P	Y
	*qTBGWb3*	3	26,908,494	36,413,819	YJCSSL-3.2.4a	19P, 20P	J
	*qTBGWb4*	4	23,402,532	27,675,276	YJCSSL-4.2.2a	19P	J
	*qTBGWb6*	6	2,002,736	21,248,529	YJCSSL-6.2, YJCSSL-6.6, YJCSSL-6.14	19P	J
	*qTBGWb7*	7	8,671,562	18,964,912	YJCSSL-7.2.3	19P	J
	*qTBGWb8*	8	1	5,104,572	YJCSSL-8.3a	19P, 20P	Y
	*qTBGWb9*	9	1	8,781,870	YJCSSL-9.1, YJCSSL-9.5, YJCSSL-9.6, YJCSSL-9.7	19P, 20P	J
BGL	*qBGLb1*	1	1	9,872,404	YJCSSL-1.1	19P, 20P	J
	*qBGLb2*	2	15,833,180	35,937,250	YJCSSL-2.12	19P	Y
	*qBGLb3.1*	3	15,733,146	23,906,861	YJCSSL-3.1.5	19P, 20P	J
	*qBGLb3.2*	3	23,671,316	26,908,494	YJCSSL-3.1.7	19P	Y
	*qBGLb3.3*	3	26,908,494	36,413,819	YJCSSL-3.2.4a	19P	J
	*qBGLb4.1*	4	1	8,137,472	YJCSSL-4.1.2	19P	J
	*qBGLb4.2*	4	17,234,547	23,402,532	YJCSSL-4.1.5	19P	Y
	*qBGLb4.3*	4	27,470,300	31,428,042	YJCSSL-4.2.2a, YJCSSL-4.3.4a	19P, 20P	J
	*qBGLb5.1*	5	1,111,445	7,957,285	YJCSSL-5.2, YJCSSL-5.5	19P, 20P	J
	*qBGLb5.2*	5	8,019,584	29,958,434	YJCSSL-5.5	19P	Y
	*qBGLb6*	6	2,002,736	21,248,529	YJCSSL-6.2, YJCSSL-6.6	19P, 20P	J
	*qBGLb7.1*	7	3,359,562	4,899,924	YJCSSL-7.2.1	19P, 20P	Y
	*qBGLb7.2*	7	8,671,562	18,964,912	YJCSSL-7.2.3	19P, 20P	J
	*qBGLb8*	8	1	5,104,572	YJCSSL-8.3a	19P	Y
	*qBGLb9*	9	1	8,781,870	YJCSSL-9.1, YJCSSL-9.5	19P, 20P	J
	*qBGLb10.1*	10	10,761,946	16,460,614	YJCSSL-10.2.3a	19P	J
	*qBGLb10.2*	10	20,489,591	23,207,287	YJCSSL-10.2.6	19P	J
	*qBGLb11*	11	1	6,231,196	YJCSSL-11.1	19P	Y
	*qBGLb12*	12	18,005,156	21,449,199	YJCSSL-12.16, YJCSSL-12.19a	19P, 20P	J
BGWI	*qBGWbI1*	1	34,385,285	43,270,923	YJCSSL-1.10	19P, 20P	Y
	*qBGWbI2*	2	15,833,180	35,937,250	YJCSSL-2.12	19P, 20P	Y
	*qBGWIb3.1*	3	1	10,077,921	YJCSSL-3.1.3	19P, 20P	Y
	*qBGWIb3.2*	3	26,908,494	36,413,819	YJCSSL-3.2.4a	20P	J
	*qBGWIb4*	4	1	8,137,472	YJCSSL-4.1.2	19P, 20P	Y
	*qBGWIb5*	5	1,111,445	7,957,285	YJCSSL-5.2, YJCSSL-5.5	19P, 20P	Y
	*qBGWIb6*	6	21,248,529	31,248,787	YJCSSL-6.14	20P	J
	*qBGWIb8*	8	5,652,612	16,939,665	YJCSSL-8.14	20P	J
	*qBGWIb9*	9	1	8,781,870	YJCSSL-9.1, YJCSSL-9.6, YJCSSL-9.7	20P	J
	*qBGWIb10*	10	1	12,667,708	YJCSSL-10.1.3a	19P, 20P	Y
BGT	*qBGTb1*	1	34,385,285	43,270,923	YJCSSL-1.10	19P, 20P	Y
	*qBGTb3*	3	26,908,494	36,413,819	YJCSSL-3.2.4a	20P	J
	*qBGTb4.1*	4	4,475,561	19,457,371	YJCSSL-4.1.2, YJCSSL-4.1.5	19P, 20P	Y
	*qBGTb4.2*	4	34,809,722	35,502,694	YJCSSL-4.3.7a	20P	J
	*qBGTb6.1*	6	1	5,155,721	YJCSSL-6.2	20P	J
	*qBGTb6.2*	6	21,248,529	31,248,787	YJCSSL-6.14	20P	J
	*qBGTb7*	7	8,671,562	18,964,912	YJCSSL-7.2.3	19P, 20P	J
	*qBGTb8*	8	1	5,104,572	YJCSSL-8.3a	20P	Y
	*qBGTb9*	9	1	8,781,870	YJCSSL-9.1, YJCSSL-9.5, YJCSSL-9.6, YJCSSL-9.7, YJCSSL-9.4	20P	J
	*qBGTb10*	10	1	12,667,708	YJCSSL-10.1.3a	19P, 20P	Y
S	*qSb3*	3	26,908,494	36,413,819	YJCSSL-3.2.4a	19P, 20P	Y
	*qSb8*	8	4,063,287	8,643,075	YJCSSL-8.3a, YJCSSL-8.14	19P, 20P	Y
Mo	*qMob3*	3	26,908,494	36,413,819	YJCSSL-3.2.4a	20P	Y
Cu	*qCub3*	3	26,908,494	36,413,819	YJCSSL-3.2.4a	19P, 20P	Y
	*qCub6*	6	1	5,155,721	YJCSSL-6.2	19P, 20P	J
	*qCub9*	9	11,624,154	20,851,851	YJCSSL-9.6, YJCSSL-9.7, YJCSSL-9.4	19P, 20P	J
Zn	*qZnb8*	8	4,063,287	8,643,075	YJCSSL-8.3a, YJCSSL-8.14	19P, 20P	J
	*qZnb9*	9	1	8,781,870	YJCSSL-9.1, YJCSSL-9.5, YJCSSL-9.6, YJCSSL-9.7, YJCSSL-9.4	19P, 20P	J
